# Photocatalytic syntheses and evaluation of biological activities of rare disaccharides, 3-*O*-α-d-glucopyranosyl-d-arabinose

**DOI:** 10.1038/s41598-025-05778-4

**Published:** 2025-07-01

**Authors:** Sho Usuki, Pratiksha Babgonda Patil, Tiangao Jiang, Naoko Taki, Yuma Uesaka, Haru Togawa, Sanjay S. Latthe, Shanhu Liu, Kenji Yamatoya, Kazuya Nakata

**Affiliations:** 1 Graduate School of Bio-Applications and Systems Engineering, Tokyo University of Agriculture and Technology, 2-24-16 Naka-cho, Koganei, Tokyo 1840012 Japan; 2 Department of Applied Biological Science, Faculty of Science and Technology, Tokyo University of Science, 2641 Yamazaki, Noda, Chiba 278-0022 Japan; 3 Vivekanand College, C.S. No 2130 E Ward, Tarabai Park, Kolhapur, Maharashtra 416 003 India; 4 Henan Joint International Research Laboratory of Environmental Pollution Control Materials, Henan Key Laboratory of Polyoxometalate Chemistry, College of Chemistry and Chemical Engineering, Henan University, Kaifeng, 475004 People’s Republic of China; 5 Laboratory of Genomic Function Engineering, Department of Life Sciences, School of Agriculture, Meiji University, 1-1-1 Higashimita, Tama-Ward, Kawasaki, Kanagawa 214-8571 Japan

**Keywords:** Rare disaccharides, Photocatalytic synthesis, 3-*O*-α-d-glucopyranosyl-d-arabinose, PtCl/TiO_2_ photocatalyst, Bioactive compounds, Photocatalysis, Sustainability

## Abstract

Recently, there has been a growing interest in rare sugars due to their potential applications in functional foods and pharmaceuticals. However, sustainable production methods for these compounds remain challenging due to their high cost, lengthy production times, and environmentally harmful reagents. Herein, we report a novel photocatalytic approach for synthesizing rare disaccharides from maltose, an abundant and renewable natural resource, under mild conditions at room temperature and atmospheric pressure using light as the energy source. The photocatalytic treatment of maltose using platinum compound-supported titanium oxide (PtCl/TiO₂) resulted in the formation of rare disaccharides, primarily 3-*O*-α-d-glucopyranosyl-d-arabinose and glucosyl-erythrose, which were characterized by HPLC, LC/MS, ^13^C NMR spectroscopy, and optical rotation measurements. Notably, biological evaluation of 3-*O*-α-d-glucopyranosyl-d-arabinose using HeLa and HEK293 cells demonstrated no cytotoxicity and negligible cellular uptake. Furthermore, enzymatic degradation studies using mouse intestinal α-glucosidase revealed significantly lower degradability compared to maltose, with minimal glucose production observed. These findings suggest that 3-*O*-α-d-glucopyranosyl-d-arabinose exhibits resistance to digestion and absorption in mammalian systems, highlighting its potential application as a low-calorie sweetener and a functional food ingredient. This study presents an environmentally benign synthetic route to rare disaccharides and demonstrates their promising biological properties.

**Supplementary Information:**

The online version contains supplementary material available at 10.1038/s41598-025-05778-4.

## Introduction

Rare sugars are monosaccharides and their derivatives that exist in limited quantities in nature^1^, representing a unique class of carbohydrates with significant potential in biomedical applications and functional food development^2–4^. These compounds have garnered attention due to their diverse physiological activities and therapeutic potential. For instance, d-allose exhibits antioxidant properties through eliminating reactive oxygen species^5^, demonstrating protective effects against oxidative stress-induced cellular damage^6^. d-Tagatose acts as a competitive inhibitor of glucose absorption in the small intestine ^7^, while d-psicose effectively competes with glucose and fructose for absorption^8^. Both rare sugars suppress postprandial glucose elevation and show promise in diabetes management^9,10^.

Despite their promising biological activities, practical implementation of rare sugars faces substantial challenges. Their extremely low natural abundance makes direct extraction economically unfeasible, presenting a significant barrier to both industrial-scale production and comprehensive investigation of their physiological functions. Consequently, developing efficient and sustainable production technologies for rare sugars has become a critical focus in the field.

Current methodologies for rare sugar synthesis include enzymatic and chemical approaches^11–13^, each with distinct advantages and limitations. Enzymatic synthesis has emerged as a promising strategy, particularly with recent discoveries of novel isomerases and epimerases that facilitate stereoselective isomerization of abundant sugars into rare sugars^13–15^. However, enzymatic methods face challenges including high enzyme production costs, gradual loss of catalytic activity, and limited operational stability under industrial conditions.

Chemical synthesis methods offer alternatives but present their own challenges^16–18^. The Ruff degradation reaction enables controlled carbon chain modification but requires potentially hazardous reagents such as bromine and ferric salts^19,20^. The Seyferth-Gilbert homologation provides means for carbon chain extension^21^, while the formose reaction enables synthesis of various sugars from formaldehyde^22^. However, these chemical methods typically involve harsh reaction conditions and environmentally harmful reagents, often resulting in complex product mixtures requiring extensive purification.

Among naturally abundant disaccharides, sucrose (glucose-fructose α1 → 2 glycosidic linkage), lactose (glucose-galactose β1 → 4 glycosidic linkage), and maltose (two glucose molecules with α1 → 4 glycosidic linkage) are particularly prominent^23^. In contrast, “rare disaccharides” constitute a unique subset characterized by their limited natural occurrence and distinctive physiological activities. Turanose (glucose-fructose α1 → 3 glycosidic linkage) exhibits α-glucosidase inhibitory activity^24^. Isomaltulose (glucose-fructose α1 → 6 glycosidic linkage) demonstrates prebiotic properties^25^. Melibiose (galactose-glucose α1 → 6 glycosidic linkage) shows selective growth-promoting effects on lactic acid bacteria^26^, while kojibiose (two glucose molecules with α1 → 2 glycosidic linkage) enhances bifidobacterial proliferation^27,28^. These distinctive physiological activities arise from their unique structural features, particularly their glycosidic linkage patterns and constituent monosaccharide combinations.

Photocatalytic conversion has emerged as a promising approach to synthesize rare sugars from biomass-derived monosaccharides under environmentally benign conditions. Photocatalysts generate electron–hole pairs upon light irradiation, facilitating oxidation and reduction reactions on their surfaces^29,30^. Recent studies have demonstrated the versatility of photocatalytic systems for monosaccharide conversions^31,32^, with various modified titanium oxide catalysts effectively converting glucose to arabinose through selective oxidation^33,34^. While these studies have established the potential for monosaccharide conversion, the application to more complex carbohydrates, particularly disaccharides, remains largely unexplored.

In this study, we investigated the photocatalytic transformation of disaccharides for the synthesis of rare sugars under ambient conditions using platinum compound-supported titanium oxide (PtCl/TiO_2_) as a photocatalyst. Using maltose as a model substrate, we investigated its photocatalytic transformation pathway, characterized the reaction products, and evaluated their biological properties through cell viability assays and enzyme-based degradation studies.

## Experimental section

### Degradation of maltose by PtCl/TiO_2_ photocatalyst

d-Maltose monohydrate (Wako Pure Chemical Industries, Ltd.) (1.8 g) was added to 50 mL of ultrapure water to prepare a 100 mM maltose solution. PtCl/TiO_2_ (MPT623, Ishihara Sangyo Kaisha, Ltd.) (20 mg) was then added to the solution. The solution was irradiated with UV light (intensity: 10 mW cm^-2^) under stirring. At specified time intervals, 1 mL aliquots of the solution were collected, the photocatalyst was separated by centrifugation, and the supernatant was recovered and filtered through a syringe filter.

### Analysis by HPLC and LC/MS

Maltose in the reaction mixture was analyzed using HPLC equipped with a refractive index detector (RI) (Shimadzu Corporation, Japan). The analysis was performed using a Sugar-D column (250 mm × 4.6 mm, 5 μm particle size, COSMOSIL) with 75% acetonitrile/25% water (v/v) as the mobile phase at a flow rate of 1.0 mL min^-1^ at 30 °C. Maltose concentrations were determined using a calibration curve.

Products of the degradation of maltose by PtCl/TiO_2_ were analyzed using HPLC (Shimadzu Corporation, Japan) or LC/MS (LC-MS8050, Shimadzu Corporation, Japan). Prior to measurement, some products were derivatized with p-aminobenzoic acid ethyl ester (ABEE) using the following procedure: ABEE solution was prepared by mixing 332.4 mg ABEE, 31.7 mg sodium cyanoborohydride, 386.4 μL acetic acid, and 3.6 mL methanol. Then, 10 μL of the product solution was mixed with 40 μL of the ABEE solution and vortexed. After centrifugation for 30 s, the mixture was heated to 80 °C. After 1 h, the mixture was cooled to room temperature and centrifuged for 30 s. Next, 200 μL of water and 200 µL of chloroform were added to the mixture and vortexed for 1 min. The supernatant (150 µL) was extracted and diluted three-fold with pure water to prepare the sample. This solution was used for HPLC or LC/MS analysis.

For HPLC analysis of ABEE-labeled samples, a CAPCELL PAK C18 column (250 mm × 4.6 mm, Shiseido) was used with either 87% ammonium acetate solution (20 mmol L^-1^)/13% acetonitrile (v/v) or 94% ammonium acetate solution (20 mmol L^-1^)/6% acetonitrile (v/v) as the mobile phase at a flow rate of 1.0 mL min^-1^ at 40 °C. Non-labeled samples were analyzed under the same conditions as those used for maltose analysis. The product concentrations were determined using calibration curves.

For LC/MS analysis, ABEE-labeled samples were analyzed using a CAPCELL PAK C18 column (250 mm × 2.0 mm, Shiseido) with 87% ammonium acetate solution (20 mmol L^-1^)/13% acetonitrile (v/v) as the mobile phase at a flow rate of 0.2 mL min^-1^ at 40 °C. Non-labeled samples were analyzed using a Sugar-D column (250 mm × 4.6 mm, 5 μm particle size, COSMOSIL) with 75% acetonitrile/25% water (v/v) as the mobile phase at a flow rate of 0.5 mL min^-1^ at 30 °C. Molecular weight determination was performed using the positive ESI mode.

### ^13^C-NMR and optical rotation measurements

The following experiments were conducted to isolate the target disaccharides for ^13^C-NMR and optical rotation measurements. d-Maltose monohydrate (1.8 g) was added to 50 mL of ultrapure water to prepare a 100 mM maltose solution. PtCl/TiO_2_ (20 mg) was then added to the solution. After the 72 h photocatalytic reaction, all the reaction solutions were filtered through a 0.22 μm nylon filter to remove PtCl/TiO_2_ from the suspension. The sample was concentrated to 5 mL using a rotary evaporator and then separated by preparative HPLC. The separation was performed using a Sugar-D preparative column (250 mm × 20 mm, COSMOSIL) with either 85% acetonitrile/15% water (v/v) or 80% acetonitrile/20% water (v/v) as the mobile phase at a flow rate of 18 mL min^-1^ at 40 °C. The samples were collected in the retention time of target products. The solvent in the collected samples was evaporated using a rotary evaporator, followed by drying under nitrogen flow. Multiple batches of the reaction solution were collected and concentrated using a rotary evaporator. The dried samples were dissolved in deuterated solvent for NMR analysis. ^13^C NMR spectra were recorded at 125 MHz using a BRUKER AVANCE/DRX-600 spectrometer.

The specific optical rotation of the isolated sample was also measured. The collected samples were dried using a rotary evaporator until a syrup-like consistency was achieved, and then dissolved in ultrapure water. The samples were subjected to ion exchange using an anion-exchange resin, followed by vacuum concentration using a rotary evaporator. Next, 5 mL of ethanol was added and mixed, followed by vacuum concentration using a rotary evaporator. This process was repeated several times. Subsequently, 5 mL of toluene was added to the samples and vacuum concentrated to evaporate the remaining water, yielding a dry white powder. This powder was dissolved in ultrapure water at a concentration of 2 mg mL^-1^, transferred to a cell, and the specific optical rotation was measured using a P1010 polarimeter (10 mm × 10 mm × 45 mm quartz cell, sodium lamp, JASCO).

### Cell culture

HeLa (human cervical cancer cells) and HEK293 (human embryonic kidney cells) cells were used in this study. HeLa cells were cultured in D-MEM/Ham’s F-12 (Wako Pure Chemical Industries) supplemented with 10% FBS (SERUM SOURCE INTERNATIONAL) and 1% penicillin–streptomycin solution (Nacalai Tesque). HEK293 cells were cultured in D-MEM (Wako Pure Chemical Industries) supplemented with 10% FBS (SERUM SOURCE INTERNATIONAL) and 1% penicillin–streptomycin solution (Nacalai Tesque). The cells were cultured in a CO_2_ incubator (Thermo Fisher Scientific) at 37 °C and 5% CO_2_. As HeLa cells are adherent cells, 1 mM-EDTA trypsin (Nacalai Tesque) was used to detach them from the culture dishes.

### Evaluation of sugar effects on cell proliferation

The effects of various sugars on HeLa cell proliferation were investigated. Hela cells were trypsinized and the cell count was adjusted to 4.0 × 10^5^ cells mL^-1^ using D-MEM/Ham’s F-12 supplemented with 10% FBS and 1% penicillin–streptomycin solution. The cell suspension was dispensed into 96-well plates (100 µL per well). After attachment of HeLa cells, the culture medium was removed using an aspirator. The glucose-free D-MEM (Wako Pure Chemical Industries) was dispensed into tubes (1 mL each), and glucose, arabinose, maltose, sucrose, or 3-*O*-α-d-glucopyranosyl-d-arabinose was added to each tube to a final concentration of 15 mM. The different sugars solutions were dispensed to HeLa cells in 96-well plates (100 µL per well). Two additional 96-well plates were prepared in a similar manner. Cell concentrations were measured using the MTT assay. Of the three prepared plates, one was immediately subjected to the MTT assay (0-h sample), while the remaining two were incubated for 24 or 48 h before the MTT assay. The MTT assay was performed as follows: after cell culture, the sugar-containing medium was removed by aspiration, and 100 µL of D-MEM was added to each well to resuspend the cells. MTT solution (10 µL) was then added, and the plates were incubated at 37 °C for one hour. After removing the supernatant, 100 µL of DMSO were added to dissolve the formazan dyes, and the absorbances were measured at 580 nm.

To determine whether the various sugars were absorbed by HeLa or HEK cells, the sugar concentrations in the culture medium were measured. HeLa or HEK cells were suspended in FBS-free, glucose-free D-MEM at 1.0 × 10^6^ cells mL^-1^ and added to 6 mL dishes. Maltose, glucose, or 3-*O*-α-d-glucopyranosyl-d-arabinose were added to a final concentration of 15 mM. Culture medium (3 mL) was collected before culture, and after 24 and 48 h of culture.

To remove proteins and cell debris from the collected culture medium, trichloroacetic acid (Nacalai Tesque) was added to a final concentration of 10% and kept on ice for 30 min. After centrifugation at 12,000 rpm, the supernatant was collected. Diethyl ether (5 mL) was added to the supernatant and vortexed for 2 min. The lower aqueous layer was collected, and its pH was measured. The process of adding diethyl ether to remove trichloroacetic acid was repeated until the aqueous layer reached a neutral pH. After complete removal of trichloroacetic acid, chloroform (5 mL) was added and vortexed for 3 min. The aqueous layer was collected to remove diethyl ether dissolved in chloroform. This sample was analyzed by HPLC to determine the sugar concentrations. All sugar analyses were performed under the same HPLC conditions as those described for the maltose analysis.

### Sugar degradation experiments using mouse intestinal α-glucosidase

The small intestine was excised from the male mice after 24 h of fasting. The excised intestine was cut open lengthwise and washed with ice-cold physiological saline. The washed intestine was transferred to a 15 mL centrifuge tube, and 12 mL of ice-cold 0.1 mM phosphate buffer (pH 7.0) containing 0.05% FBS were added, followed by homogenization. After homogenization, the mixture was centrifuged at 1,500 × g for 5 min to remove intestinal debris. The supernatant was used as the α-glucosidase solution for subsequent experiments.

Maltose, sucrose, or 3-*O*-α-d-glucopyranosyl-d-arabinose was dissolved in 0.1 M phosphate buffer to a final concentration of 15 mM. These sugar solutions were mixed with equal volumes (500 µL each) of α-glucosidase solution and incubated at 37 °C for 30 min. After incubation, trichloroacetic acid was added to a final concentration of 10% to stop the α-glucosidase reaction, and the mixture was kept on ice for 30 min. Subsequently, trichloroacetic acid was removed using diethyl ether, and the diethyl ether was removed using chloroform. This sample was analyzed by HPLC to determine sugar concentrations in the aqueous solution.

This study was approved and conducted according to the guidelines for the care and use of laboratory animals of the Tokyo University of Science (approval No. N18011). We confirmed that all experiments in this study were performed in accordance with the relevant guidelines and regulations. All the procedure of the study is followed by the ARRIVE guidelines.

### Enzyme inhibition assay

A solution containing maltose (final concentration, 5 mM) and 3-*O*-α-d-glucopyranosyl-d-arabinose (final concentration, 20 mM) was prepared. This mixed solution (250 µL) was combined with an equal volume of α-glucosidase solution extracted from the mouse intestine, as described above. The mixture was then incubated at 37 °C for 10 min. After incubation, trichloroacetic acid was added to a final concentration of 10%. Subsequent processing was performed following the same procedure as the sugar degradation experiments with α-glucosidase, and the maltose concentration was measured by HPLC under the same conditions as the maltose analysis described above. As a control, a sample containing only 5 mM maltose mixed with the α-glucosidase solution was also prepared and analyzed for maltose concentration under the same HPLC conditions.

## Results and discussion

### Photocatalytic conversion of maltose using PtCl/TiO_2_

**Fig. 1:**
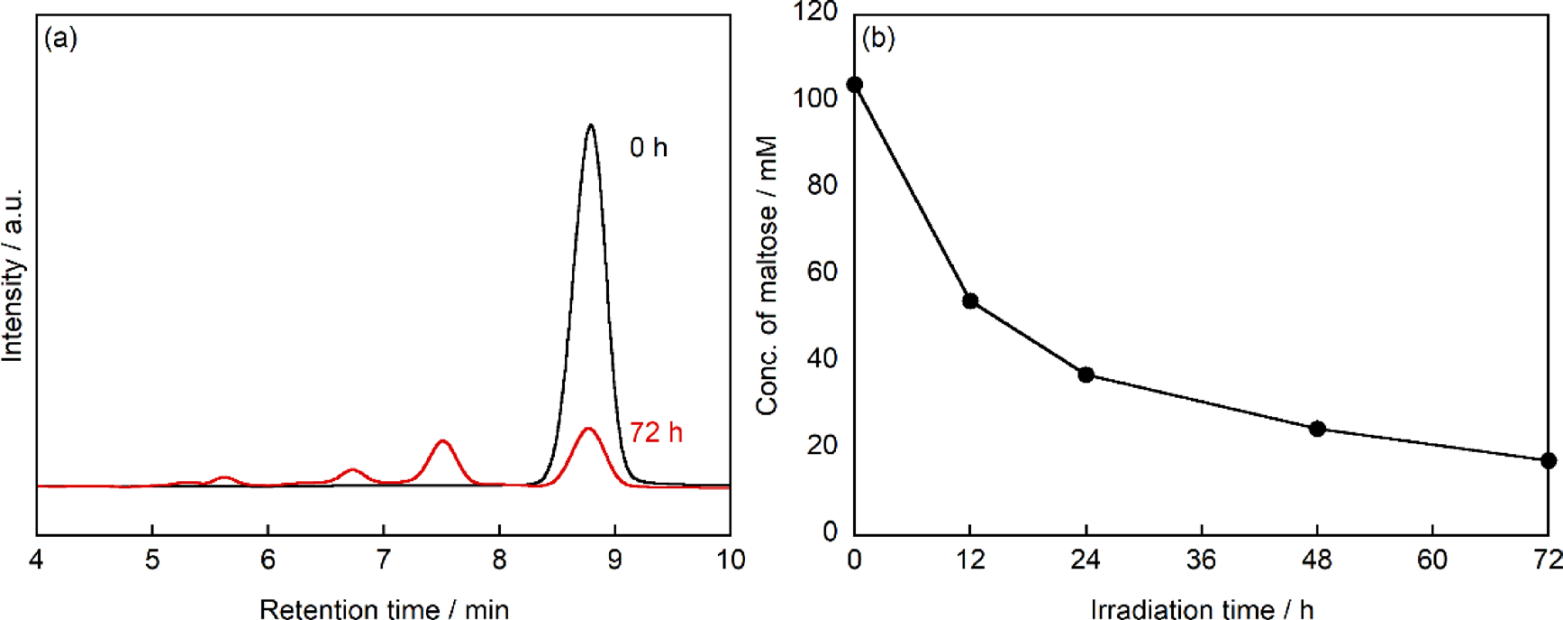
(**a**) HPLC chromatograms for the sample obtained before (black line) and after (red line) the photocatalytic treatment of maltose for 72 h under UV irradiation. Column: Sugar-D, mobile phase: 75% acetonitrile/25% water (v/v) at a flow rate of 1.0 mL min^-1^ at 30 °C, detector: RI. (**b**) Changes in maltose concentration as a function of irradiation time. Maltose: 100 mmol L^-1^, PtCl/TiO_2_ photocatalyst: 20 mg, UV light: 10 mW cm^-2^, temperature: 25 ℃.

The photocatalytic transformation of maltose was investigated using PtCl/TiO₂ under UV irradiation at room temperature in air. HPLC analysis of the reaction mixture after 72 h revealed several characteristic peaks (Fig. 1a). The peak at retention time (R.T.) of 8.8 min corresponded to unreacted maltose, as confirmed by comparison with an authentic standard. The intensity of this peak decreased significantly after 72 h of UV irradiation compared with that of the initial sample. The time course of maltose concentration, determined from the HPLC peak areas, showed a gradual decrease from 104 to 18.4 mmol L^−1^ over the 72 h reaction period (Fig. 1b). The time course of maltose concentration, determined from the HPLC peak areas, showed a gradual decrease from 104 to 18.4 mmol L^−1^ after 72 h (Fig. 1b).

The primary product peak observed at R.T. = 7.5 min was analyzed by LC/MS to investigate molecular weight. Subsequent LC/MS analysis of the product revealed characteristic signal at *m/z* = 335 and 351 (Fig. S1a). The consistent mass differences of 16 between these signals correspond to the typical mass differences between the [M + Na]⁺, and [M + K]⁺ adducts, which are commonly observed in electrospray ionization mass spectrometry. Furthermore, the product peak observed at R.T. = 7.5 min (Fig. 1a) was isolated via preparative HPLC and derivatized with p-aminobenzoic acid ethyl ester (ABEE) to enhance chromatographic separation. Subsequent LC/MS analysis of the ABEE-derivatized product revealed three characteristic signals at *m/z* = 462, 484, and 500 (Fig. S1b). The consistent mass differences of 22 and 16 between these signals correspond to the typical mass differences between the [M + H]⁺, [M + Na]⁺, and [M + K]⁺ adducts, which are commonly observed in electrospray ionization mass spectrometry. Accounting for the molecular weight of the ABEE label (149) and the respective ionizing species (H⁺: 1, Na⁺: 23, and K⁺: 39), the molecular weight of the underivatized product was calculated to be 312. This molecular weight is consistent with that of a disaccharide structure containing one hexose and one pentose unit joined by a glycosidic linkage.

**Fig. 2:**
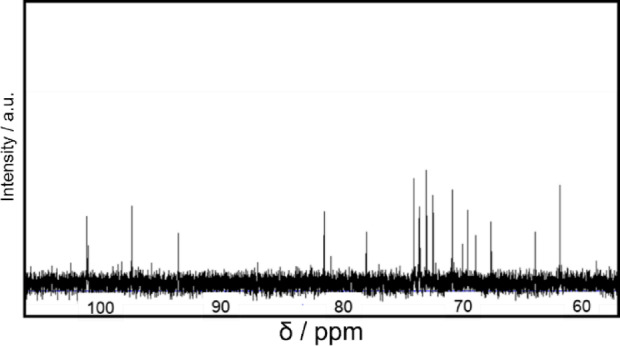
^13^C NMR spectrum of obtained 3-*O*-α-d-glucopyranosyl-d-arabinose. Maltose: 100 mmol L^-1^, PtCl/TiO_2_ photocatalyst: 20 mg, UV light: 10 mW cm^-2^, temperature: 25 °C.

To elucidate the structure of the isolated product, ^13^C NMR spectroscopy was performed (Fig. 2). Since 3-*O*-α-d-glucopyranosyl-d-arabinose has limited reported examples and commercially available standards are difficult to obtain, qualitative analysis was conducted by comparison with previous literature reports. The chemical shifts are in excellent agreement with those previously reported for 3-*O*-α-d-glucopyranosyl-d-arabinose in the literature (Table S1)^35^. This spectral assignment, combined with the molecular weight of 312 determined by LC/MS analysis, unambiguously identified the product as 3-*O*-α-d-glucopyranosyl-d-arabinose, a disaccharide comprising glucose and arabinose moieties linked via an α-glycosidic bond.

The optical rotation of the isolated product was measured as additional structural characterization. The specific rotation was determined to be [α]_D_ =  + 50.4°, whose value is similar to previously reported specific rotations ([α]_D_ =  + 47.0° to + 56.9°) for 3-*O*-α-d-glucopyranosyl-d-arabinose under comparable conditions. The consistency between our measured optical rotation and literature values provides additional confirmation of the product’s structural assignment^36^. Together with the spectroscopic data from LC/MS and ^13^C NMR analyses, these results unambiguously establish that the photocatalytic treatment of maltose generates 3-*O*-α-d-glucopyranosyl-d-arabinose.

**Fig. 3:**
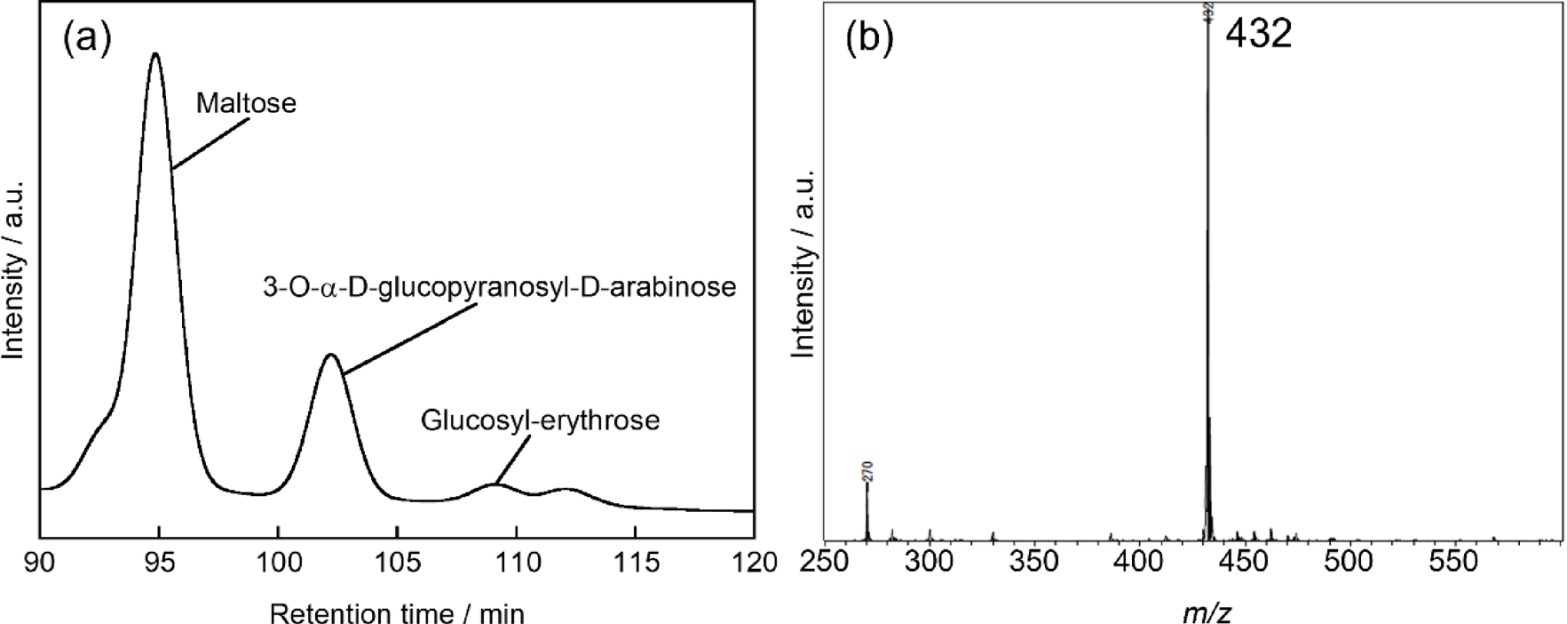
(**a**) HPLC chromatogram obtained using a LC/MS system with a UV–VIS detector for the sample obtained after PtCl/TiO_2_ treatment of maltose under UV irradiation for 72 h. The products were functionalized using ABEE. Column: CAPCELL PAK C18, mobile phase: 94% ammonium acetate solution (20 mmol L-1)/6% acetonitrile (v/v) at a flow rate of 0.1 mL min^-1^ at 40 °C. (**b**) Mass spectrum of the isolated sample found in R.T. = 109.1 min in HPLC analysis.

Further analysis of the reaction products was performed after 72 h of photocatalytic treatment. The ABEE-derivatized samples were analyzed by LC/MS, resulting in the separation of three distinct peaks: maltose (R.T = 94.9 min), 3-*O*-α-d-glucopyranosyl-d-arabinose (R.T = 102.2 min), and an additional product (R.T = 109.1 min) (Fig. 3a). Mass spectrometric analysis of the component eluted at R.T = 109.1 min revealed an m/z value of 432 (Fig. 3b). Considering the mass contribution of the ABEE label (149) and proton adduct (H⁺: 1), the molecular weight of the underivatized product was determined to be 282. This molecular weight corresponds to that of glucosyl-erythrose, which contains one less carbon atom than 3-*O*-α-d-glucopyranosyl-d-arabinose.

**Fig. 4:**
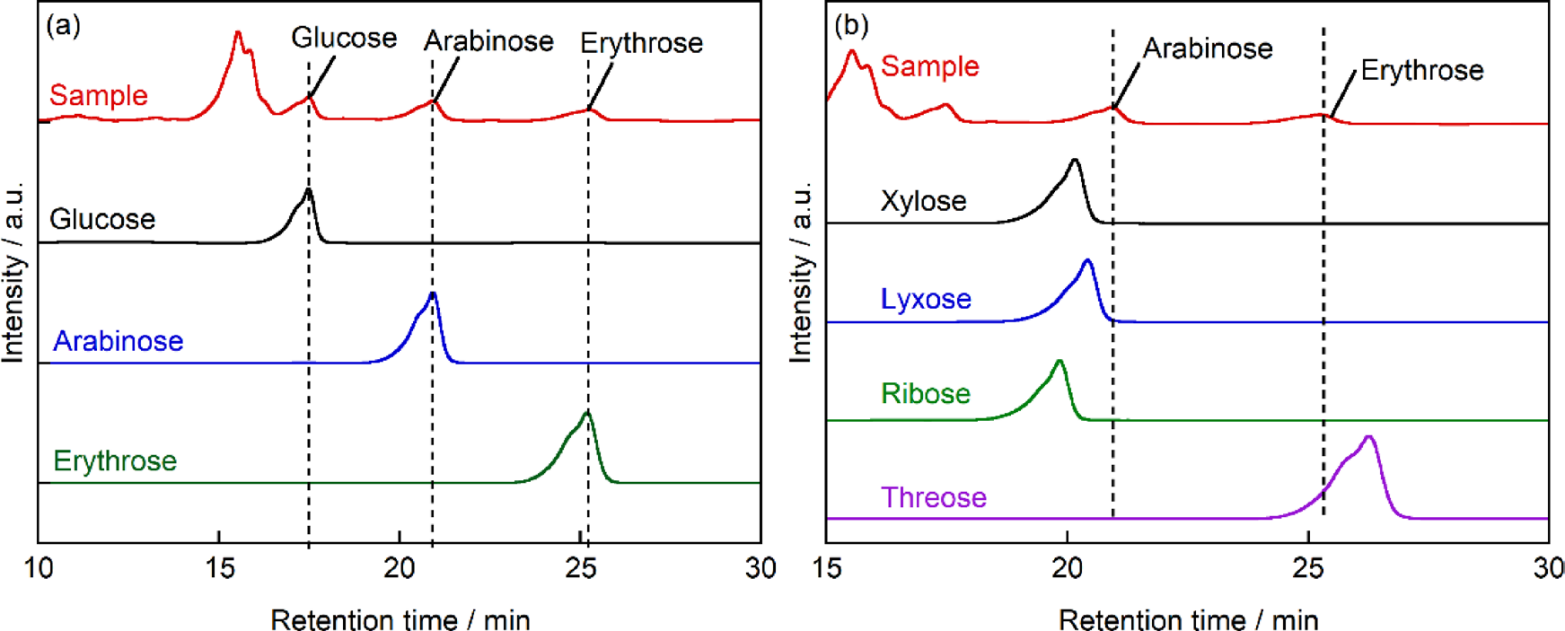
HPLC chromatograms for the sample obtained after photocatalytic treatment of maltose. The sample was compared with (**a**) standards of the identified monosaccharides and (**b**) alternative monosaccharide standards to confirm structural assignments. All products were derivatized with ABEE prior to analysis. Column: CAPCELL PAK C18, mobile phase: 87% ammonium acetate solution (20 mmol L^-1^)/13% acetonitrile (v/v) at a flow rate of 1.0 mL min^-1^ at 40 °C.

HPLC analysis of the ABEE-derivatized sample after 72 h of photocatalytic treatment revealed additional product peaks at retention times of 17.5, 20.9, and 25.3 min (Fig. 4a). These retention times were compared with those of authentic standards: glucose (hexose), arabinose (pentose), and erythrose (tetrose). The peaks at R.T. = 17.5, 20.9, and 25.3 min corresponded to those of glucose, arabinose, and erythrose standards, respectively. The molecular weights of these components were further confirmed by LC/MS analysis. The peak at R.T. = 17.5 min exhibited three signals at m/z = 330, 352, and 368, corresponding to the [M + H]⁺, [M + Na]⁺, and [M + K]⁺ adducts, respectively (Fig. S2(a)). Based on these mass spectral data, considering the ABEE label (149) and ionizing species (H⁺: 1, Na⁺: 23, K⁺: 39), the molecular weight was calculated to be 180. Similarly, LC/MS analysis of the peaks at R.T. = 20.9 and 25.3 min yielded molecular weights of 150 and 120, respectively (Figs. S2(b) and (c)). These molecular weights match those of glucose, arabinose, and erythrose, confirming the formation of these monosaccharides during the photocatalytic reaction. Furthermore, R.T. of the ABEE-derivatized sample after 72 h of photocatalytic treatment were compared with those of additional monosaccharide standards: xylose, lyxose, and ribose (all pentoses), as well as threose (a tetrose) (Fig. 4b). The R.T. of all peaks in the sample did not correspond to any of these standard monosaccharides, further confirming that the specific monosaccharides formed during the photocatalytic reaction were arabinose and erythrose.

**Fig. 5:**
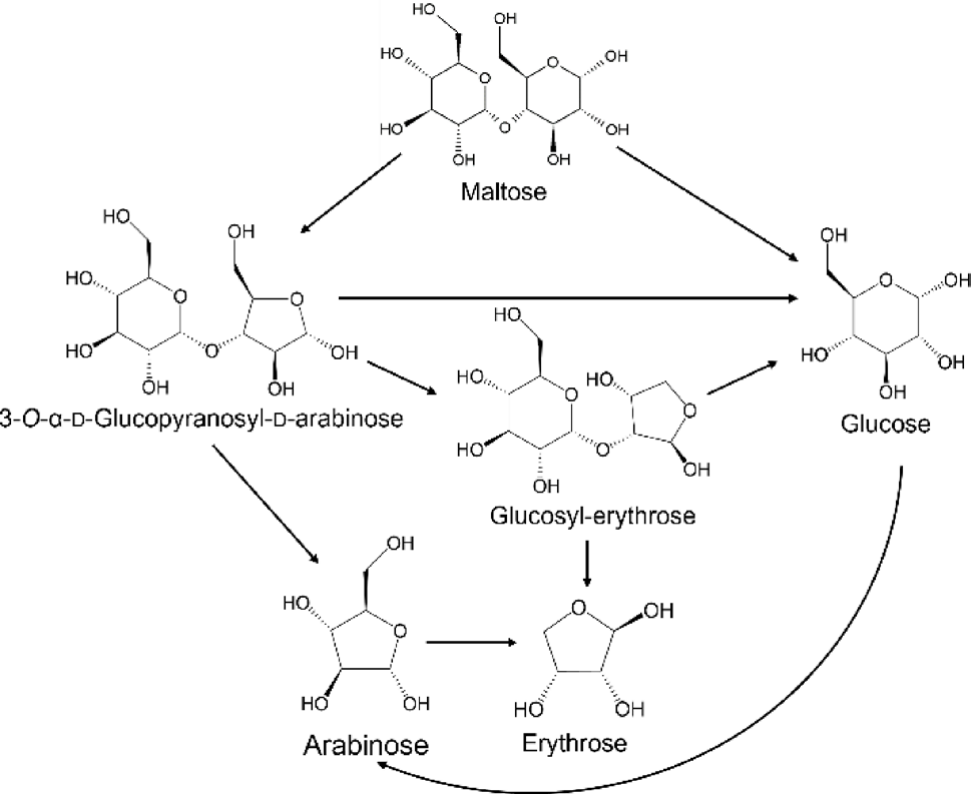
Plausible reaction pathway of decomposition of maltose and generation of products using PtCl/TiO_2_ photocatalyst under light irradiation. The α-anomeric forms are drawn at all reducing ends of sugars.

The analytical results demonstrated that the photocatalytic treatment of maltose with PtCl/TiO₂ under UV irradiation at room temperature in air led to the formation of 3-*O*-α-d-glucopyranosyl-d-arabinose, glucosyl-erythrose, glucose, arabinose, and erythrose. Two distinct reaction pathways are proposed for this transformation (Fig. 5): (1) decarbonylation of the terminal aldehyde group and (2) glycosidic bond cleavage.

The decarbonylation pathway is supported by previous studies demonstrating the photocatalytic conversion of glucose to arabinose using TiO₂-based catalysts^37^. In aqueous solution, maltose exists in equilibrium between its closed-ring and open-ring forms, with the latter being more susceptible to photocatalytic oxidation. The observed formation of 3-*O*-α-d-glucopyranosyl-d-arabinose can be attributed to the photocatalytic decarbonylation of the C1 carbon in maltose. Further decarbonylation likely leads to the formation of glucosyl-erythrose.

The glycosidic bond cleavage pathway appears to proceed through hydroxyl radical-mediated mechanisms. Hydroxyl radicals, generated by the photocatalyst, can cleave glycosidic bonds through both direct and indirect pathways, as reported by Peshev *et al*^38^. The direct pathway involves a hydroxyl radical attack on the C-H bond adjacent to the glycosidic bond in the glucose segment^38^. Alternatively, according to DFT calculations by Dai et al., the indirect pathway is initiated by a radical attack at C6, generating a carbon radical^39^. Subsequently, the C6-OH hydrogen migrates to the pyran ring oxygen via a seven-membered-ring transition state mediated by a water molecule^39^. This process leads to pyran ring opening and formation of an unstable hemiacetal structure at the glycosidic bond. The final cleavage occurs through decarbonylation of the C-1 carbon of the reducing glucose entity in maltose.

A control experiment was conducted to examine whether acidic conditions contributed to glycosidic bond cleavage, given that the reaction solution reached a pH of 2 after 72 h. The maltose solution containing PtCl/TiO₂ was adjusted to pH 2 using formic acid and monitored for 72 h. No glucose formation was detected, indicating that the observed glycosidic bond cleavage was primarily due to photocatalytically generated hydroxyl radicals rather than acid-catalyzed hydrolysis.

It has been reported that sugars predominantly exist in cyclic structures in aqueous solutions, with the open-chain forms constituting less than 1%. Therefore, it is considered that maltose and the sugar products in this study primarily exist in cyclic structures^40^. The glucose generated through glycosidic bond cleavage subsequently undergoes photocatalytic decarbonylation to form arabinose, which is further converted to erythrose. Additionally, both 3-*O*-α-d-glucopyranosyl-d-arabinose and glucosyl-erythrose can undergo glycosidic bond cleavage to yield their respective monosaccharide components. These reaction pathways are summarized in Fig. 5.

The yields of each product calculated from the HPLC chromatogram after 72 h of degradation are shown in Table 1. The highest yield was for 3-*O*-α-d-glucopyranosyl-d-arabinose, followed by d-glucose. This suggests that among the two decomposition pathways discussed above, the decarbonylation reaction occurs more frequently than the glycosidic bond cleavage, or that the decarbonylation reaction rate of the generated d-glucose is relatively high. Additionally, other products not listed in the table may include organic acids, aldehydes, and the final decomposition product, carbon dioxide. The carbon balance of products after the 72-h photocatalytic reaction was analyzed by HPLC. The results showed that 36.1% of the carbon content from the initial maltose was obtained as the target disaccharide, while 52.9% was identified as characterized byproducts. The remaining 11% was detected as trace components.

**Table 1:** Conversion yields of each products.

Products	Conversion yield (%)
3-*O*-α-d-glucopyranosyl-d-arabinose	26.1
Glucosyl-erythrose	2.2
Glucose	12.9
Arabinose	6.9

### Evaluation of biological activities of 3-*O*-α-d-glucopyranosyl-d-arabinose effects

**Fig. 6:**
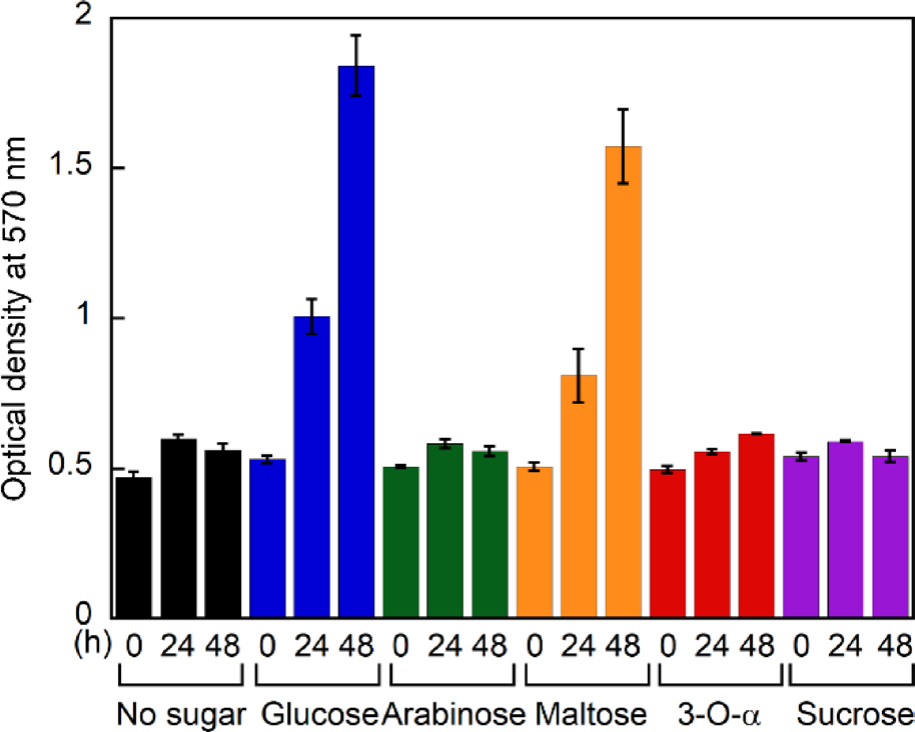
Changes in absorbance measured by MTT assay in the presence of HeLa cells and various sugars (n = 3).

The biological effects of 3-*O*-α-d-glucopyranosyl-d-arabinose were evaluated using HeLa cells through MTT assays. Cells were cultured in media supplemented with various sugars (glucose, maltose, arabinose, sucrose, or 3-*O*-α-d-glucopyranosyl-d-arabinose) at a final concentration of 15 mM, and cell proliferation was monitored for 48 h (Fig. 6). In glucose-free medium, no increase in absorbance was observed, indicating suppression of cell proliferation. In contrast, glucose supplementation resulted in a significant increase in absorbance, indicating active cell proliferation. Maltose supplementation also promoted cell proliferation, albeit to a lesser extent than glucose supplementation. Cells cultured with arabinose or sucrose showed no increase in absorbance, consistent with previous reports, indicating that HeLa cells lack specific transporters for these sugars. Similarly, cells cultured with 3-*O*-α-d-glucopyranosyl-d-arabinose exhibited no increase in absorbance. Notably, although arabinose, sucrose, and 3-*O*-α-d-glucopyranosyl-d-arabinose did not support cell proliferation, no decrease in absorbance was observed under any of these conditions, indicating the absence of cytotoxicity. 3-*O*-α-d-glucopyranosyl-d-arabinose has lower reactivity than maltose. This is possibly due to structural differences between these disaccharides. These results suggest that 3-*O*-α-d-glucopyranosyl-d-arabinose, which is not utilized for cell proliferation, exhibits no apparent cytotoxicity toward HeLa cells.

The MTT assay quantifies viable cells by measuring the absorbance of formazan, produced through the reduction of MTT (3-(4,5-Dimethyl-2-thiazolyl)-2,5-diphenyltetrazolium bromide) by mitochondrial reductases in metabolically active cells^41^. In this study, cells cultured in glucose-free medium showed no increase in absorbance, while glucose supplementation resulted in a progressive increase in absorbance during the 48 h incubation period. This observation is consistent with the known characteristics of HeLa cells, which exhibit high glucose uptake capacity to support their proliferation. Similarly, maltose supplementation led to a time-dependent increase in absorbance during the 48 h culture period, indicating its ability to support cell proliferation. In contrast, 3-*O*-α-d-glucopyranosyl-d-arabinose, similar to arabinose and sucrose, showed no change in absorbance throughout the 48 h experiment, suggesting that these sugars neither supported cell proliferation nor exhibited cytotoxicity. 3-*O*-α-d-glucopyranosyl-d-arabinose has lower reactivity possibly than maltose. This is due to structural differences between these disaccharides.

**Fig. 7:**
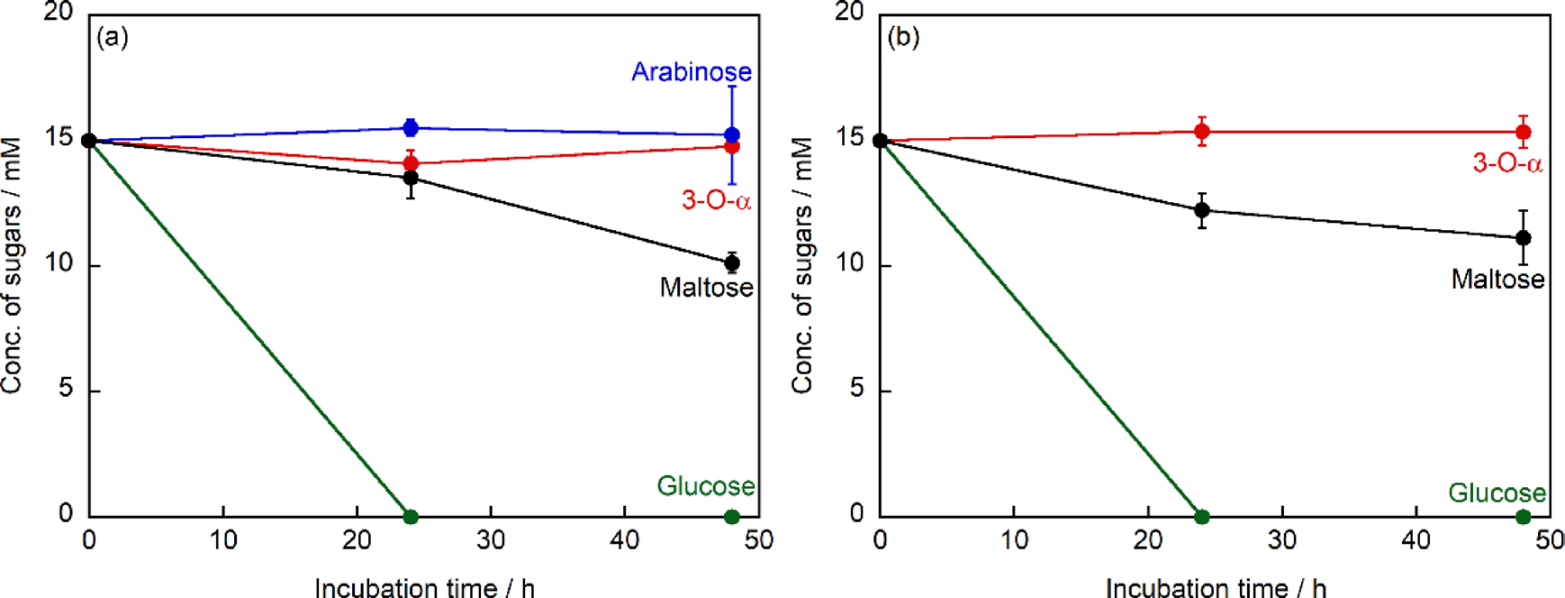
Changes in sugar concentrations in (**a**) HeLa cell, (**b**) HEK293 cell culture supernatants (n = 3).

To investigate the cellular uptake of 3-*O*-α-d-glucopyranosyl-d-arabinose, we quantitatively analyzed sugar concentrations in the culture supernatant of HeLa cells using HPLC. Temporal changes in extracellular concentrations of various sugars (glucose, maltose, arabinose, and 3-*O*-α-d-glucopyranosyl-d-arabinose) were monitored during 48 h of incubation (Fig. 7a). In glucose-supplemented cultures, extracellular glucose concentration exhibited a time-dependent decrease, reaching undetectable levels after 24 h. This observation aligns with the well-documented glucose utilization characteristics of HeLa cells. Similarly, the maltose concentration in the culture supernatant decreased progressively during the 48-h incubation period, albeit at a slower rate compared to that of glucose. The observed decrease in maltose concentration, coupled with the cell proliferation data, suggests its utilization by the cells. In marked contrast, the concentration of 3-*O*-α-d-glucopyranosyl-d-arabinose remained constant throughout the 48-h incubation period, with no significant deviation from the initial concentration of 15 mM (*p* > 0.05). A similar pattern was observed for arabinose, which maintained a stable extracellular level. These findings indicate that neither 3-*O*-α-d-glucopyranosyl-d-arabinose nor arabinose underwent significant cellular uptake or extracellular degradation. This interpretation is consistent with the cell proliferation data, which showed no growth-promoting effect of 3-*O*-α-d-glucopyranosyl-d-arabinose.

To examine whether the observed uptake patterns were cell type-specific, we extended our investigation to HEK293 cells, a non-cancerous human cell line derived from embryonic kidney tissue. Sugar concentrations in the culture supernatant were analyzed under experimental conditions identical to those used for HeLa cells. Analysis of the culture supernatant during 48 h of incubation revealed that both glucose and maltose concentrations progressively decreased (Fig. 7b). This pattern of glucose and maltose utilization was comparable to that observed in HeLa cells, suggesting similar sugar uptake characteristics between the two cell lines. Significantly, the concentration of 3-*O*-α-d-glucopyranosyl-d-arabinose in the HEK293 culture supernatant remained constant at 15 mM throughout the 48-h incubation period (*p* > 0.05). This result demonstrates that the lack of cellular uptake of 3-*O*-α-d-glucopyranosyl-d-arabinose is not restricted to HeLa cells but extends to non-cancerous human cells. 3-*O*-α-d-glucopyranosyl-d-arabinose has lower reactivity possibly than maltose. This is due to structural differences between these disaccharides.

**Fig. 8:**
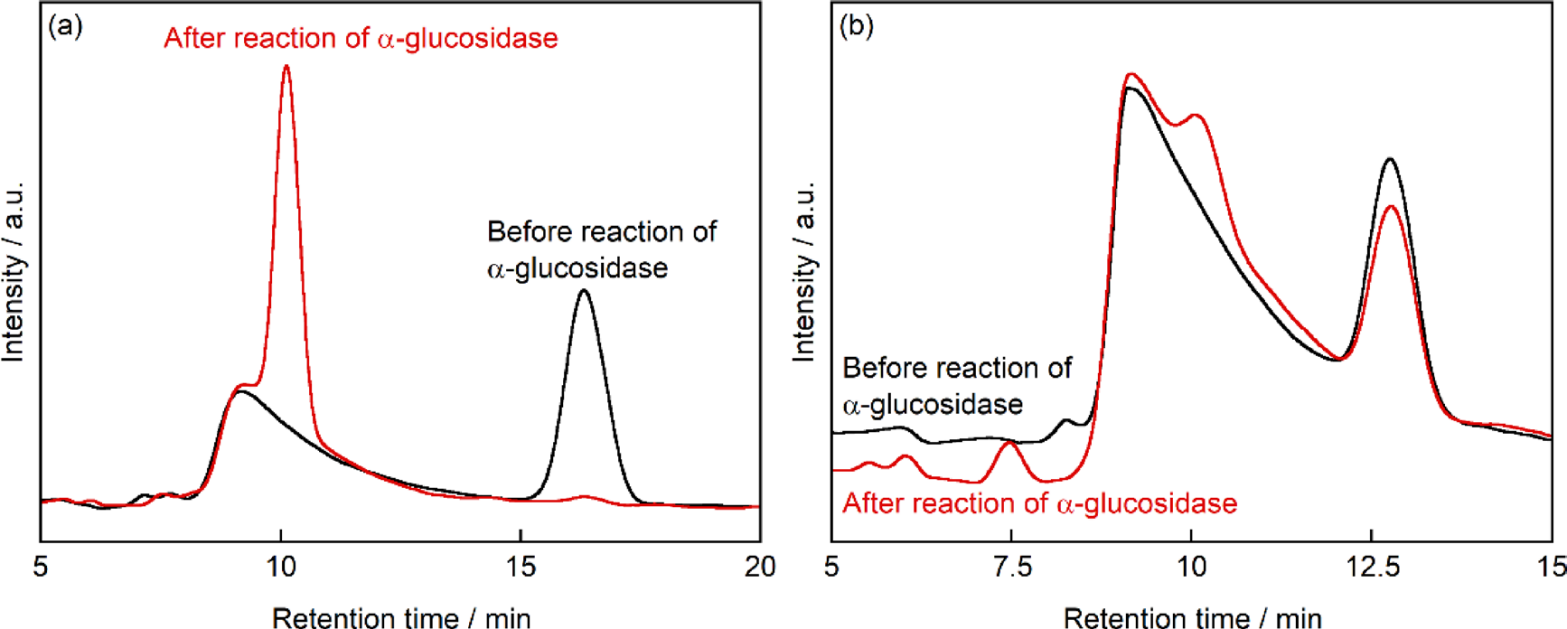
HPLC chromatogram for the sample obtained before (black line) and after (red line) reaction of (**a**) maltose or (**b**) 3-*O*-α-d-glucopyranosyl-d-arabinose with α-glucosidase for 30 min (n = 3). Column: Sugar-D, mobile phase: 75% acetonitrile/25% water (v/v) at a flow rate of 1.0 mL min^-1^ at 30 °C, detector: RI.

Based on the observation that 3-*O*-α-d-glucopyranosyl-d-arabinose exhibited minimal cellular uptake, we investigated its susceptibility to enzymatic degradation to evaluate its potential as a bioactive compound. Initially, we assessed the activity of mouse intestinal α-glucosidase using maltose as a model substrate. The enzyme solution was incubated with maltose (15 mM) at 37 °C for 30 min, followed by protein precipitation with trichloroacetic acid and HPLC analysis of the supernatant. HPLC analysis revealed that in samples containing both maltose and α-glucosidase, the characteristic maltose peak (R.T. = 16.3 min) disappeared, concurrent with the appearance of a new peak at R.T. = 10.1 min, corresponding to glucose (Fig. 8a). This observation confirmed the enzymatic hydrolysis of maltose to glucose by mouse intestinal α-glucosidase, thus validating the activity of our enzyme preparation. The peak at R.T. = 9 min was observed in both samples containing either maltose or 3-*O*-α-d-glucopyranosyl-d-arabinose, as evidenced in Fig. 8. This peak appeared consistently both before and after enzymatic treatment, indicating it does not originate from products of enzymatic processing. It is noteworthy that all samples contained enzyme solution extracted from mouse intestine. Although this enzyme solution predominantly contains α-glucosidase, it is not completely purified. Consequently, the solution likely contains additional components beyond α-glucosidase. We postulate that these components were detected as the peak at R.T. = 9 min. Control experiments without α-glucosidase showed no conversion of maltose, demonstrating that the observed hydrolysis was enzyme-dependent.

Having confirmed the activity of mouse intestinal α-glucosidase, we investigated the enzymatic degradation of 3-*O*-α-d-glucopyranosyl-d-arabinose under identical experimental conditions. HPLC analysis of the reaction mixture revealed two notable changes after 30 min of incubation (Fig. 8b). First, the peak corresponding to 3-*O*-α-d-glucopyranosyl-d-arabinose (R.T. = 12.8 min) showed a modest decrease in intensity compared to that of the control sample without α-glucosidase. Second, a new peak appeared at R.T. = 10.1 min, corresponding to glucose, indicating partial hydrolysis of the glycosidic bond. Quantitative analysis revealed marked differences in the degradation rates of the two disaccharides. While maltose concentration decreased substantially from 15 to 3.2 mM after 30 min of enzymatic treatment, 3-*O*-α-d-glucopyranosyl-d-arabinose exhibited only minimal degradation, with concentration decreasing from 15 to 14 mM under identical conditions. This significant difference in susceptibility to enzymatic hydrolysis suggests that 3-*O*-α-d-glucopyranosyl-d-arabinose has lower reactivity than maltose with mouse intestinal α-glucosidase, possibly due to structural differences between these disaccharides.

The differential metabolic responses to maltose and 3-*O*-α-d-glucopyranosyl-d-arabinose observed in our cellular experiments provide significant insights into their biological processing. When cells were cultured with maltose, we observed both cellular proliferation and a concurrent decrease in extracellular maltose concentration. Given that human cells lack specific maltose transporters, these observations suggest that maltose undergoes extracellular enzymatic hydrolysis, with the resulting glucose being actively transported into cells to support proliferation. This interpretation is consistent with the known presence of membrane-associated α-glucosidases in human cells.

In marked contrast, 3-*O*-α-d-glucopyranosyl-d-arabinose exhibited remarkably different biological behavior. The constant extracellular concentration throughout the culture period, coupled with the absence of cellular proliferation, strongly indicates that this compound neither undergoes significant extracellular degradation nor cellular uptake. This biological inertness can be attributed to the high substrate specificity of mammalian carbohydrate-processing enzymes. Indeed, our enzymatic studies with mouse intestinal α-glucosidase quantitatively demonstrated this specificity difference: maltose was rapidly hydrolyzed with its concentration decreasing from 15 to 3.2 mM within 30 min, whereas 3-*O*-α-d-glucopyranosyl-d-arabinose showed minimal degradation (15 mM to 14 mM). These findings suggest that 3-*O*-α-d-glucopyranosyl-d-arabinose exhibits resistance to digestion and absorption in mammalian systems. This study presents an environmentally benign synthetic route to rare disaccharides and demonstrates their fundamental biological properties, establishing a foundation for further exploration of their potential applications. The results of this study indicate that 3-*O*-α-d-glucopyranosyl-d-arabinose exhibits minimal cytotoxicity and demonstrates resistance to degradation and absorption by intestinal disaccharide-hydrolyzing enzymes. Similar to cellobiose^42^, this compound is likely to be resistant to hydrolysis by human enzymes, suggesting a significantly lower nutritional energy contribution compared to readily digestible sugars. However, unlike cellobiose, this compound possesses a unique structure composed of glucose and arabinose, potentially harboring novel biological activities and applications that await elucidation in future research. These characteristics are expected that 3-*O*-α-d-glucopyranosyl-d-arabinose holds promise as a functional food ingredient.

Notably, we observed a discrepancy between the complete absence of degradation in cell culture experiments and the slight degradation detected with mouse intestinal α-glucosidase preparations. This differential response likely reflects the distinct enzymatic environments: cellular experiments primarily involved membrane-bound enzymes, whereas the intestinal preparations contained a broader spectrum of enzymatic activities. The observed partial hydrolysis in intestinal preparations might be attributed to the substrate promiscuity of intestinal maltase-glucoamylase (MGAM)^43^, although contributions from other hydrolytic enzymes cannot be excluded. It is important to note that our α-glucosidase preparation, obtained through intestinal homogenization, contained various cellular components including lysosomal hydrolases. To definitively characterize the enzyme specificity, future studies should employ purified recombinant α-glucosidase expressed from cloned MGAM genes.

Following our observation of differential enzymatic susceptibility, we investigated the potential inhibitory effects of 3-*O*-α-d-glucopyranosyl-d-arabinose on α-glucosidase activity. This investigation was motivated by the structural similarity between 3-*O*-α-d-glucopyranosyl-d-arabinose and maltose, which differs only in the replacement of one glucose moiety with arabinose. To evaluate potential competitive inhibition, we conducted enzyme inhibition assays using a mixture of 3-*O*-α-d-glucopyranosyl-d-arabinose and maltose with mouse intestinal α-glucosidase preparation. The residual maltose concentrations were quantitatively analyzed by HPLC after enzymatic treatment. Control reactions containing maltose and α-glucosidase yielded a mean residual maltose concentration of 3.2 (95% C.I., 2.2 to 4.3) mM. In comparison, reactions containing both maltose and 3-*O*-α-d-glucopyranosyl-d-arabinose resulted in a residual maltose concentration of 4.7 (95% C.I., 4.3 to 5.0) mM. However, statistical analysis using student t-test revealed no significant differences between these conditions (*p* = 0.11, n = 3).

The present findings demonstrate that 3-*O*-α-d-glucopyranosyl-d-arabinose exhibits minimal cytotoxicity and resistance to metabolic degradation and absorption. However, several limitations in our current study necessitate further investigation. While our findings from the MTT assays and mouse intestinal α-glucosidase experiments provide valuable preliminary data, comprehensive in vivo toxicological studies are essential to establish its safety profile in animal models. Additionally, to establish its metabolic fate in humans, experiments using human-derived α-glucosidase are necessary to validate our current findings obtained from mouse enzymes. The unique properties of this compound, particularly its resistance to digestion and limited absorption in mammalian systems, suggest it may have potential applications as a functional food ingredient. These systematic evaluations are essential prerequisites for advancing 3-*O*-α-d-glucopyranosyl-d-arabinose toward practical applications in food science and nutrition.

The observed minimal absorption and degradation in mammalian systems suggest potential applications for this compound in future research. These biological properties, combined with the sustainable synthetic approach developed in this study, make 3-*O*-α-d-glucopyranosyl-d-arabinose a promising candidate for further investigation.

## Conclusions

In this study, we have demonstrated the photocatalytic transformation of maltose using PtCl/TiO₂ catalyst under mild conditions, leading to the formation of rare disaccharides. The primary product, 3-*O*-α-d-glucopyranosyl-d-arabinose, was formed through oxidative decarbonylation at the terminal aldehyde group, resulting in a glucose-arabinose glycosidic linkage structure. Further photocatalytic processing yielded glucosyl-erythrose through subsequent decarbonylation reactions. Concurrent with these transformations, we observed glycosidic bond cleavage of maltose generating glucose, which underwent further conversion to produce arabinose and erythrose. These findings establish a novel, environmentally benign synthetic route to rare disaccharides under ambient conditions. Biological evaluation of 3-*O*-α-d-glucopyranosyl-d-arabinose revealed several noteworthy characteristics. Cell viability assays demonstrated no significant cytotoxicity, while enzymatic studies using mouse intestinal α-glucosidase indicated remarkable resistance to degradation compared to conventional disaccharides. The observed minimal absorption and degradation in mammalian systems suggest potential applications as a low-calorie alternative to traditional sweeteners. These biological properties, combined with the sustainable synthetic approach developed in this study, make 3-*O*-α-d-glucopyranosyl-d-arabinose a promising candidate for future development in functional food applications. Our findings not only contribute to the expanding field of rare sugar synthesis but also demonstrate the utility of photocatalytic methods for carbohydrate transformations.

## Electronic supplementary material

Below is the link to the electronic supplementary material.

Supplementary Material 1

## Data Availability

All data included in this study is available upon request by contact with the corresponding author.
