# Supplementary material for: Photocatalytic syntheses and evaluation of biological activities of rare disaccharides, 3-O-α-d-glucopyranosyl-d-arabinose
Source: Sci Rep. 2025 Jul 1;15:21694. doi: 10.1038/s41598-025-05778-4 (PMC12215579; doi:10.1038/s41598-025-05778-4)
Supplement: Supplementary file 1 — Supplementary Material 1 [file 41598_2025_5778_MOESM1_ESM.docx]

**Supplementary information**

**Photocatalytic Syntheses and Evaluation of Biological Activities of Rare Disaccharides, 3-O-α-d-glucopyranosyl-d-arabinose**

Sho Usuki^1,2^, Pratiksha Babgonda Patil^1^, Tiangao Jiang^1^, Naoko Taki^1^, Yuma Uesaka^1^, Haru Togawa^1^, Sanjay S. Latthe^3^, Shanhu Liu^4^, Kenji Yamatoya^2,5,*^, Kazuya Nakata^1,2,*^

^1^Graduate School of Bio-Applications and Systems Engineering, Tokyo University of Agriculture and Technology, 2-24-16 Naka-cho, Koganei, Tokyo 1840012, Japan

^2^Department of Applied Biological Science, Faculty of Science and Technology, Tokyo University of Science, 2641 Yamazaki, Noda, Chiba 278-0022, Japan

^3^Vivekanand College, C.S. No 2130 E ward, Tarabai Park, 416 003 Kolhapur, Maharashtra, India

^4^Henan Joint International Research Laboratory of Environmental Pollution Control Materials, Henan Key Laboratory of Polyoxometalate Chemistry, College of Chemistry and Chemical Engineering, Henan University, Kaifeng, 475004, PR China

^5^Laboratory of Genomic Function Engineering, Department of Life Sciences, School of Agriculture, Meiji University, 1-1-1 Higashimita, Tama-ward, Kawasaki 214-8571, Kanagawa, Japan


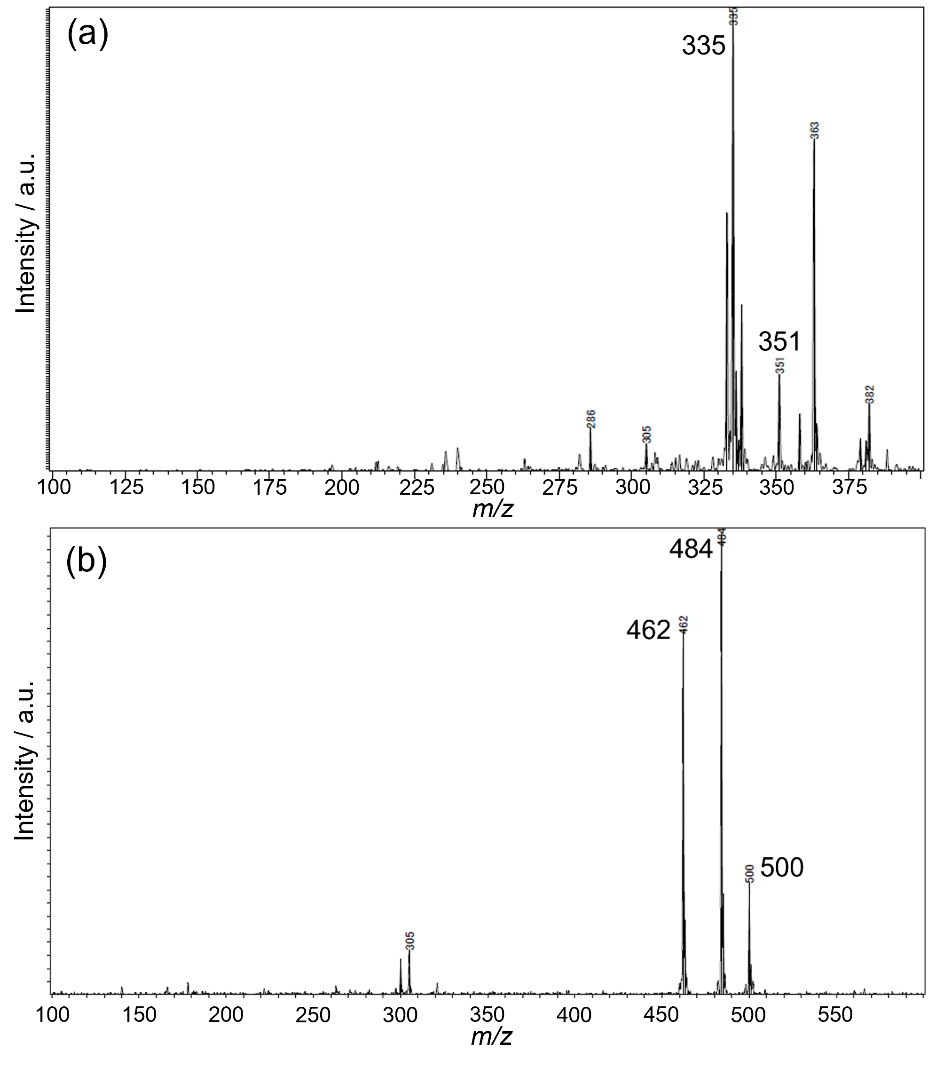


**Fig. S1.** Mass spectrum of the isolated sample found at R.T. = 7.5 min in HPLC analysis. The product was functionalized (a) without and, (b) with ABEE. Maltose: 100 mmol L^-1^, PtCl/TiO_2_ photocatalyst: 20 mg, UV light: 10 mW cm^-2^, temperature: 25 °C.


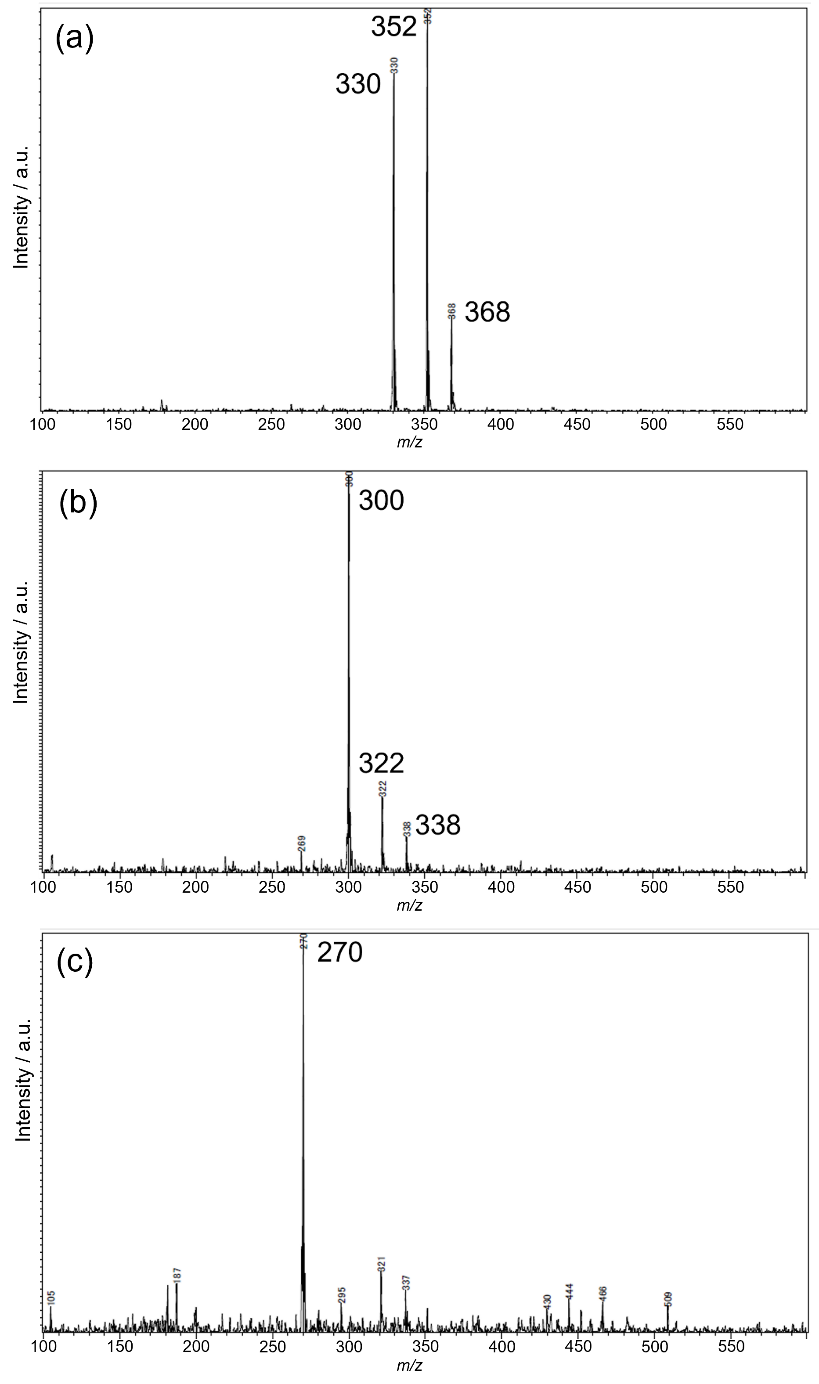


**Fig. S2.** Mass spectrum of the isolated sample found in (a) R.T. = 17.5 min, (b) R.T. = 20.9 min and (c) R.T. = 25.3 min in HPLC analysis.

**Table S1** Chemical shift of obtained 3-*O*-α-d-glucopyranosyl-d-arabinose

| atom | Literature value of  3-*O*-α-d-glucopyranosyl-d-arabinose^41^ | | Obtained 3-*O*-α-d-glucopyranosyl-d-arabinose | |
| --- | --- | --- | --- | --- |
|  | α | β | α | β |
| C-1 | 97.5 | 93.8 | 97.9 | 93.9 |
| C-2 | 73.0 | 68.8 | 73.0 | 68.9 |
| C-3 | 81.6 | 78.1 | 81.6 | 78.1 |
| C-4 | 69.5 | 69.5 | 69.5 | 69.5 |
| C-5 | 67.5 | 61.7 | 67.6 | 61.8 |
| C-1’ | 101.5 |  | 101.5 |  |
| C-2’ | 73 |  | 73 |  |
| C-3’ | 74 |  | 74.1 |  |
| C-4’ | 70.8 |  | 70.8 |  |
| C-5’ | 73.5 |  | 73.6 |  |
| C-6’ | 61.7 |  | 61.8 |  |
